# Supplementary material for: miR-27b targets MAIP1 to mediate lipid accumulation in cultured human and mouse hepatic cells
Source: Commun Biol. 2023 Jun 24;6:669. doi: 10.1038/s42003-023-05049-w (PMC10290684; doi:10.1038/s42003-023-05049-w)
Supplement: Supplementary file 2 — Supplementary Materials [file 42003_2023_5049_MOESM2_ESM.pdf]

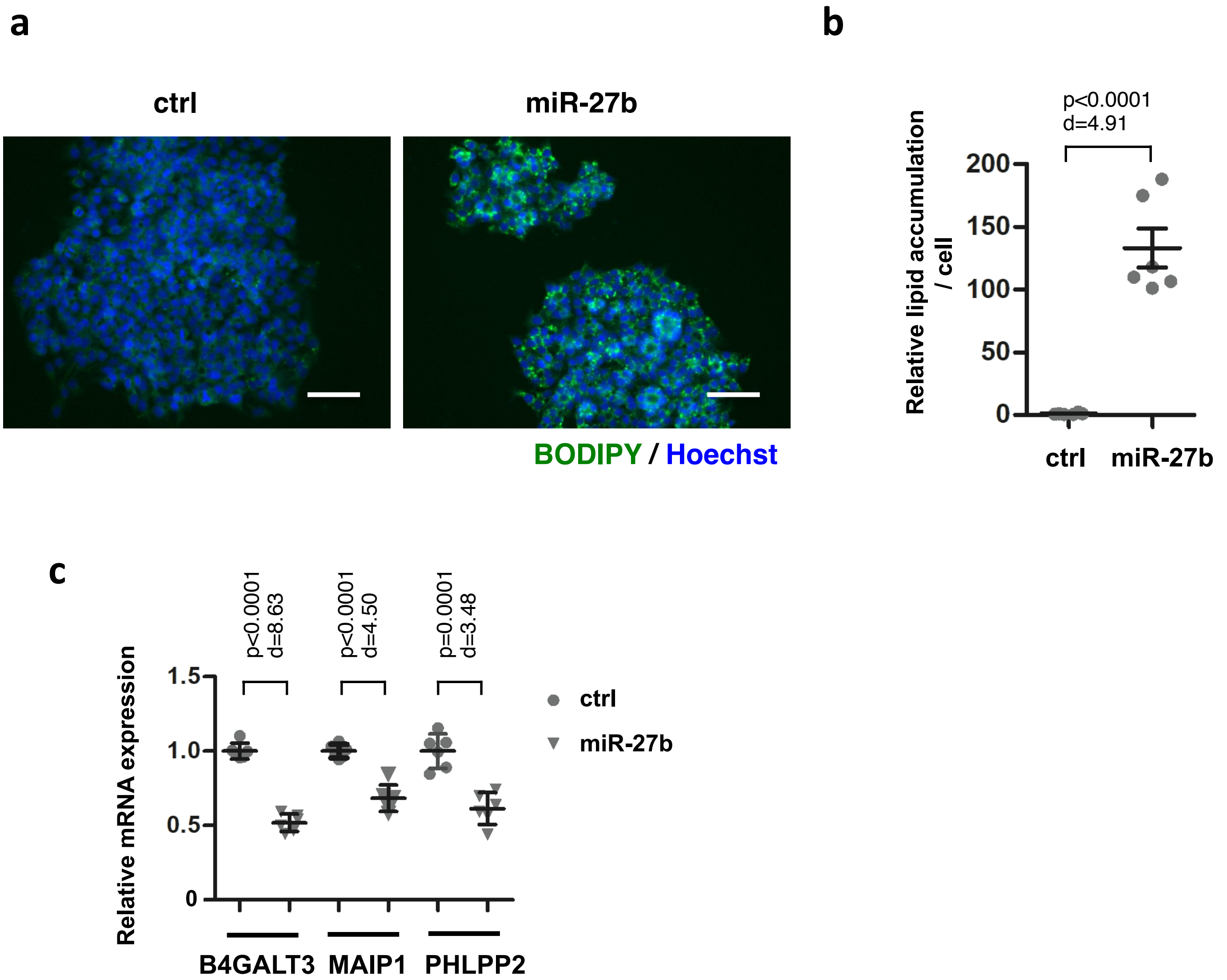

Supplementary Figure 1 *miR-27b* induces lipid accumulation in HepG2 cells. (a) HepG2 cells were transfected with control miRNA (ctrl) and *miR-27b* mimics at 10 nM followed by co-staining with BODIPY493/503 for the lipid and Hoechst 33342 for the nuclei 4 days after transfection. Scale bars, 100 mm. (b) The relative lipid amount was estimated based on the images in (a). n=3 biologically independent samples. Representative results from two independent experiments are presented as means ± SD. p-values from unpaired-2-tailed Student's t-test and Cohen's d-values are shown. (c) The mRNA levels were examined 2 days after transfection by qRT-PCRs. n=3 biologically independent samples. Representative results from two independent experiments are presented as means ± SD. p-values from unpaired-2-tailed Student's t-test and Cohen's d-values are shown. Gray circles, ctrl; gray triangles, *miR-27b* mimics.

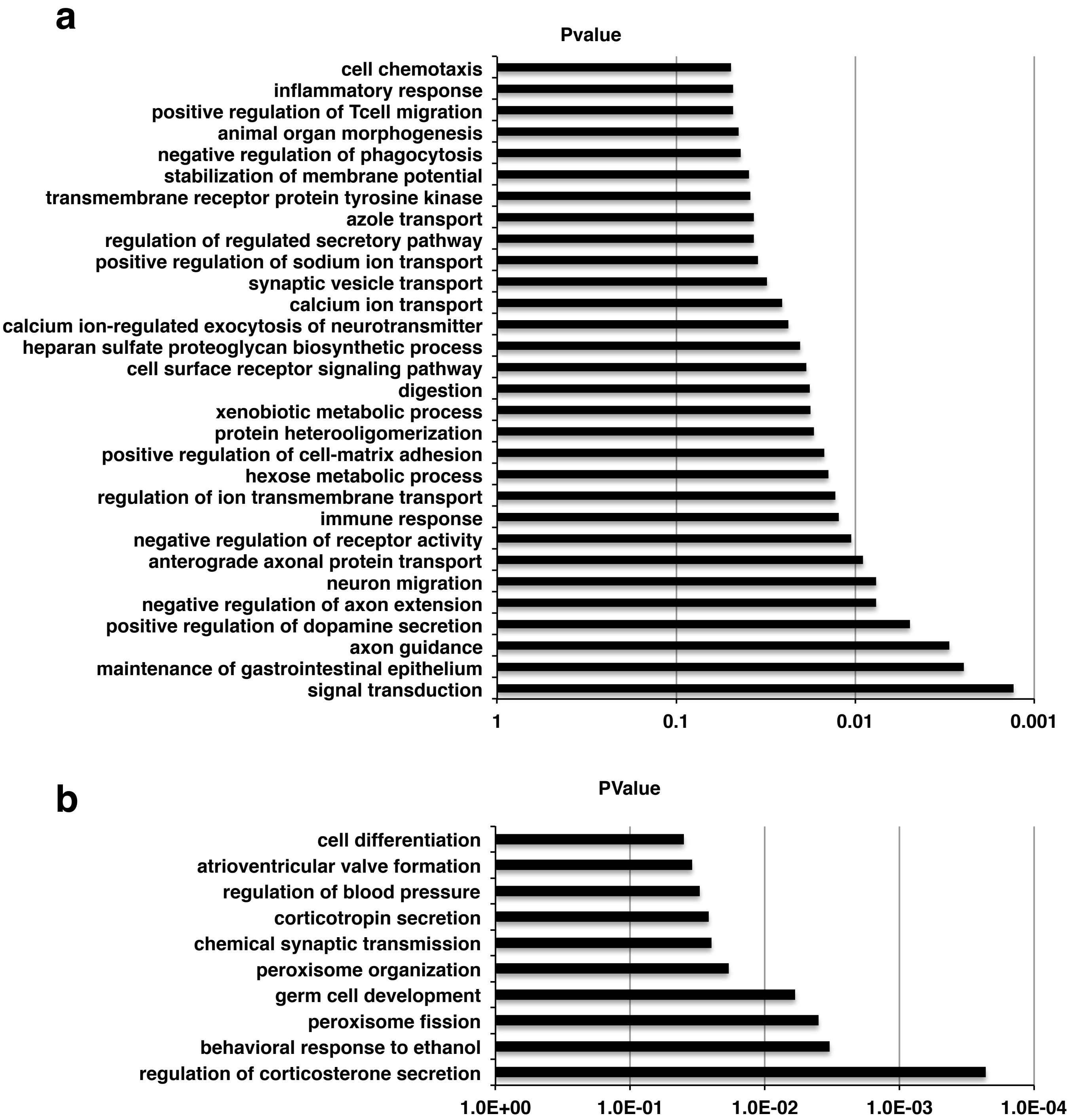

Supplementary Figure 2 Functional annotation analysis of differentially expressing genes in *miR-27b*–transfected Huh-7 cells. Genes with twofold up-(a) and down-(b) regulated expression in microarray analysis were analyzed for enrichment by DAVID (https://david.ncifcrf.gov). Pathways with significant enrichment ($p<0.5$) are shown.

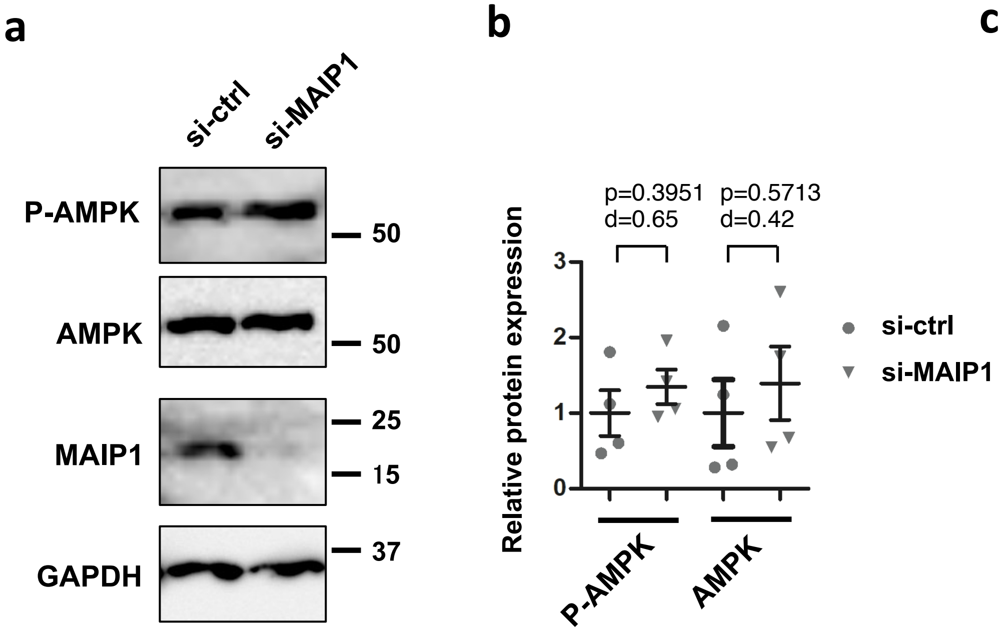

Supplementary Figure 3 ***MAIP1*-knockdown does not inactivate *AMPK*.**
(a) Huh-7 cells were transfected with control siRNA (si-ctrl) and si-*MAIP1* (10 nM) and protein expression was examined 3 days after transfection by Western blotting. Molecular weight markers are shown on the right. *GAPDH* is the internal control. (b and c) Protein expression was quantitated based on the images in (a), and relative AMPK protein expression (b) and the ratio of phosphorylated AMPK and total AMPK protein (c) are shown. n=3 independent experiments. Results are presented as means ± SE. p-values from unpaired-2-tailed Student's t-test and Cohen's d-values are shown. Gray circles, si-ctrl; gray triangles, si-MAIP1 in (b).

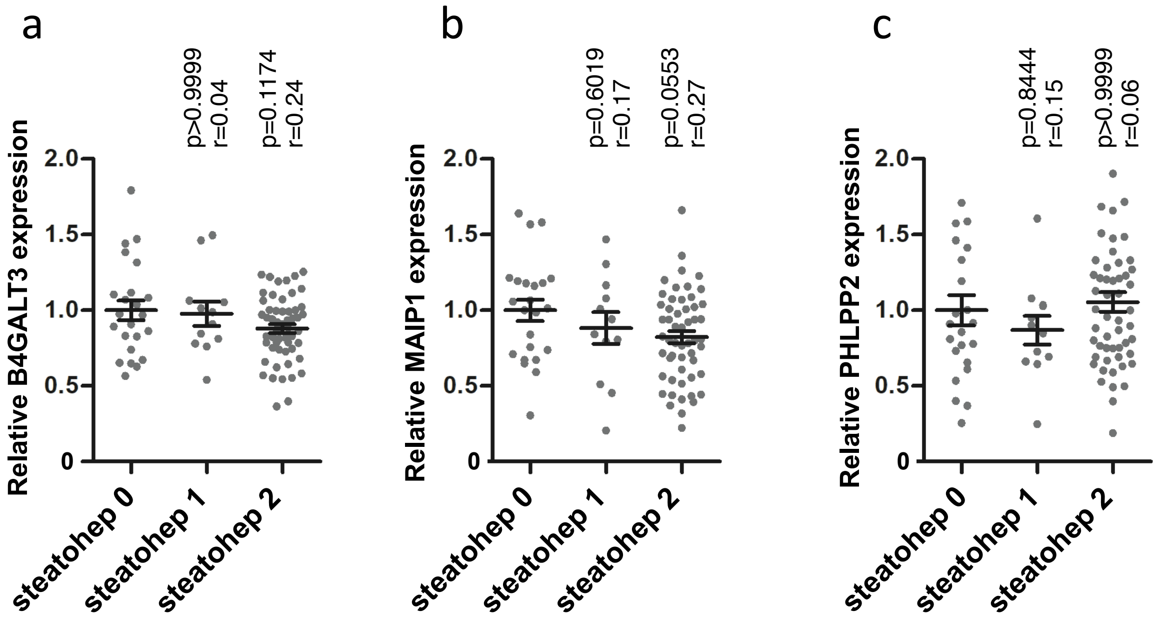

Supplemenatary Figure 4 RNA expression of *B4GALT3*, *MAIP1* and *PHLPP2* in liver biopsies from NASH patients.
Published RNA seq data (GE225740) was analyzed for *B4GALT3* (a), *MAIP1*(b) and *PHLPP2* (c). RNA count was normalized by β-actin (*ACTB*). Steatohepatitis was categorized as 0, 1, and 2, representing no steatohepatitis (n=23 independent biopsies), borderline steatohepatitis (n=12 independent biopsies) and definite steatohepatitis (n=54 independent biopsies), respectively. Relative levels to steatohepatitis 0 were presented. p-values from one way ANOVA followed by Bonferroni's post-hoc comparisons tests for steatohep 0 and Pearson's r-values are shown.

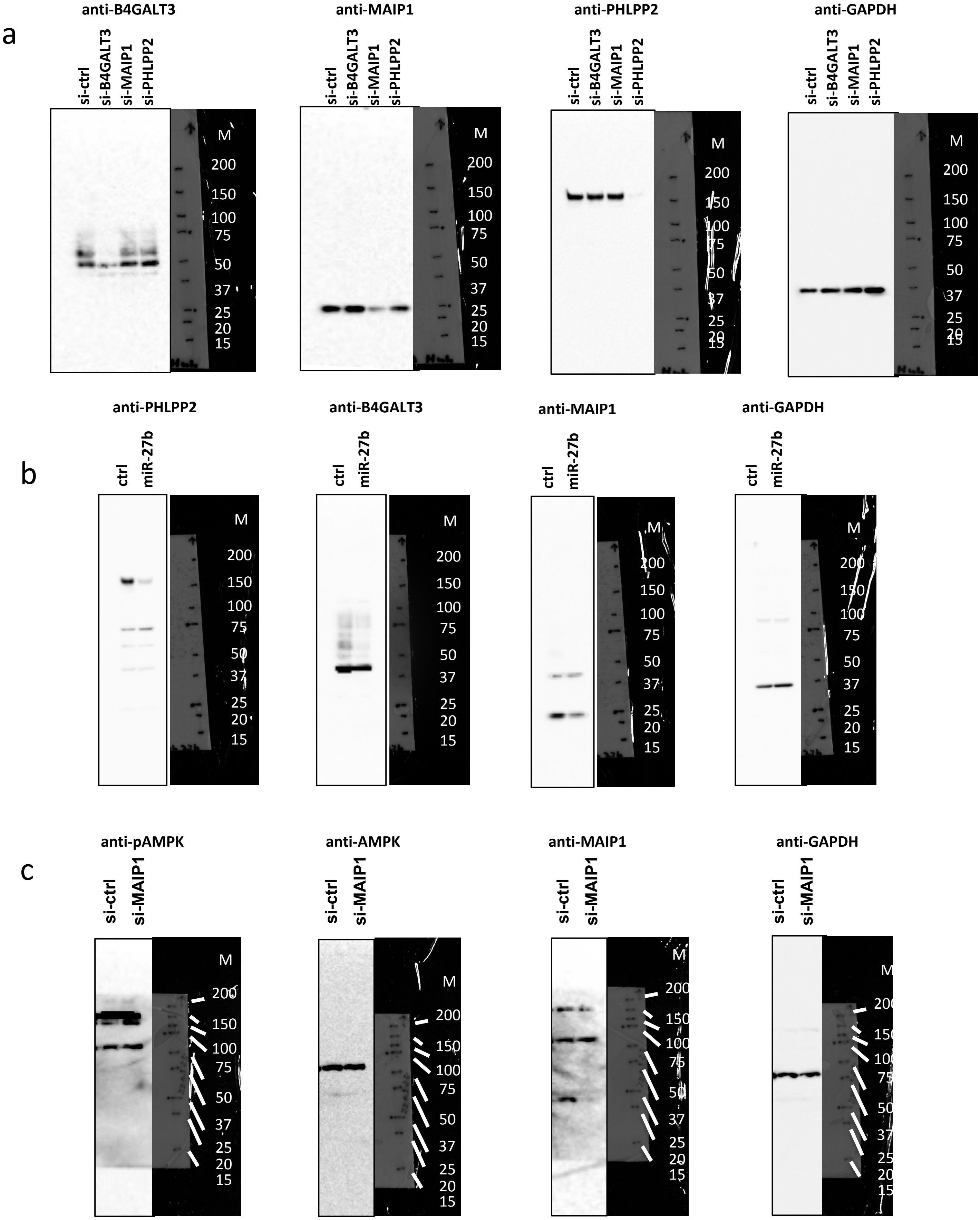

Supplementary Figure 5 Scans and blots related to Figure 3a (a), Figure 4b (b) and Supplementary Figure 3a (c).

Supplementary Table 1 Up and downregulated genes in the lipid and fatty acid-related functional annotation and KEGG pathways.

Upregulated genes	
**Functional annotation**	**Gene**
fatty acid biosynthesis	ELOVL4, FADS2
fatty acid metabolism	CYP2S1, ELOVL4, FADS2
lipid metabolism	CPS1, FABP3, FADS2, FAR2, ELOVL4, FUT1, GPAT2, CYP2S1, HSD3B1, HSD3B7, MBOAT1, PCYT1B, PIP5KL1, PLB1, PLCB2, PLCXD3, PSAPL1, TPTE2, UGT1AB
lipid byosynthesis	FADS2, FAR2, ELOVL4, GPAT2, MBOAT1, PCYT1B
lipid degradation	PLCB2, PLCXD3
Lipid transport	APOL5
phospholipid byosynthesis	GPAT2, MBOAT1, PCYT1B
phospholipid metabolism	GPAT2, MBOAT1, PCYT1B, PLB1
**KEGG pathway**	**Gene**
lipid and atherosclerosis pathway	PYCARD, HSPA6, PLCB2, TLR2
sphingolipid metabolism	PSAPL1
non-alcohlic fatty liver disease	COX7B2
fatty acid metabolism	ELOVL4, FADS2
PPAR sginaling pathway	FABP3, FADS2
**Downregulated genes**	
**Functional annotation**	**Gene**
lipid transport	STARD6
lipid metabolism	ACADL, ACER1, B4GALT3, IDI2, PIK3C2G, SULT1E1
sphingolipid metabolism	ACER1
lipid byosynthesis	IDI2
fatty acid metabolism	ACADL
**KEGG pathway**	**Gene**
lipid and atherosclerosis pathway	MIB2, CALM3, PPARG
sphingolipid metabolism	ACER1
non-alcohlic fatty liver disease	PPARG
fatty acid metabolism	ACADL
PPAR signaling pathway	ACADL, PPARG

Supplementary Table 2 The *miR-27b*-target candidate genes selected from Supplementary Data set 2 and the genes, mouse orthologs of which were also predicted as *miR-27b* targets.

Gene	Sequence Description	Accession #
ACADL	Homo sapiens acyl-CoA dehydrogenase, long chain (ACADL), nuclear gene encoding mitochondrial protein, mRNA [NM_001608]	NM_001608
AIF1L	Homo sapiens allograft inflammatory factor 1-like (AIF1L), transcript variant 4, mRNA [NM_001185096]	NM_001185096
AKIRIN1	Homo sapiens akirin 1 (AKIRIN1), transcript variant 1, mRNA [NM_024595]	NM_024595
AQP11	Homo sapiens aquaporin 11 (AQP11), mRNA [NM_173039]	NM_173039
ARF3	Homo sapiens ADP-ribosylation factor 3 (ARF3), mRNA [NM_001659]	NM_001659
ASPH	Homo sapiens aspartate beta-hydroxylase (ASPH), transcript variant 3, mRNA [NM_032466]	NM_032466
ATOH8	Homo sapiens mRNA; cDNA DKFZp761E2117 (from clone DKFZp761E2117). [AL831857]	AL831857
B4GALT3	Homo sapiens UDP-Gal:betaGlcNAc beta 1,4- galactosyltransferase, polypeptide 3 (B4GALT3), transcript variant 1, mRNA [NM_001199873]	NM_001199873
BRPF3	Homo sapiens bromodomain and PHD finger containing, 3 (BRPF3), mRNA [NM_015695]	NM_015695
C17orf103 (NATD1)	Homo sapiens chromosome 17 open reading frame 103 (C17orf103), mRNA [NM_152914]	NM_152914
C2CD2	Homo sapiens C2 calcium-dependent domain containing 2 (C2CD2), transcript variant 1, mRNA [NM_015500]	NM_015500
C2orf47 (MAIP1)	Homo sapiens chromosome 2 open reading frame 47 (C2orf47), mRNA [NM_024520]	NM_024520
CAB39L	Homo sapiens calcium binding protein 39-like (CAB39L), transcript variant 1, mRNA [NM_030925]	NM_030925
CALM3	Homo sapiens calmodulin 3 (phosphorylase kinase, delta) (CALM3), mRNA [NM_005184]	NM_005184
CBX1	Homo sapiens chromobox homolog 1 (CBX1), transcript variant 1, mRNA [NM_006807]	NM_006807
CCNG1	Homo sapiens cyclin G1 (CCNG1), transcript variant 1, mRNA [NM_004060]	NM_004060
CHIC1	Homo sapiens cysteine-rich hydrophobic domain 1 (CHIC1), mRNA [NM_001039840]	NM_001039840
CKAP4	Homo sapiens cytoskeleton-associated protein 4 (CKAP4), mRNA [NM_006825]	NM_006825
CNTLN	Homo sapiens centlein, centrosomal protein (CNTLN), transcript variant 2, mRNA [NM_001114395]	NM_001114395
CSMD1	Homo sapiens CUB and Sushi multiple domains 1 (CSMD1), mRNA [NM_033225]	NM_033225
DCHS2	Homo sapiens dachsous 2 (Drosophila) (DCHS2), transcript variant 2, mRNA [NM_001142552]	NM_001142552
DCP2	Homo sapiens DCP2 decapping enzyme homolog (S. cerevisiae) (DCP2), transcript variant 1, mRNA [NM_152624]	NM_152624
DDAH1	Homo sapiens dimethylarginine dimethylaminohydrolase 1 (DDAH1), transcript variant 1, mRNA [NM_012137]	NM_012137
DOCK8	Homo sapiens dedicator of cytokinesis 8 (DOCK8), transcript variant 1, mRNA [NM_203447]	NM_203447
DPYD	Homo sapiens dihydropyrimidine dehydrogenase (DPYD), transcript variant 1, mRNA [NM_000110]	NM_000110
EFNA5	Homo sapiens ephrin-A5 (EFNA5), mRNA [NM_001962]	NM_001962
ELF5	Homo sapiens E74-like factor 5 (ets domain transcription factor) (ELF5), transcript variant 1, mRNA [NM_198381]	NM_198381
FAM104B (TMEM29)	Homo sapiens family with sequence similarity 104, member B (FAM104B), transcript variant 1, mRNA [NM_138362]	NM_138362
FAM69A	Homo sapiens family with sequence similarity 69, member A (FAM69A), transcript variant 5, mRNA [NM_001252273]	NM_001252273
GAPT	Homo sapiens GRB2-binding adaptor protein, transmembrane (GAPT), mRNA [NM_152687]	NM_152687
GCH1	Homo sapiens GTP cyclohydrolase 1 (GCH1), transcript variant 1, mRNA [NM_000161]	NM_000161
GDF6	Homo sapiens growth differentiation factor 6 (GDF6), mRNA [NM_001001557]	NM_001001557
GPR133	Homo sapiens G protein-coupled receptor 133 (GPR133), mRNA [NM_198827]	NM_198827
GRM5	Homo sapiens glutamate receptor, metabotropic 5 (GRM5), transcript variant b, mRNA [NM_000842]	NM_000842
GXYLT1	Homo sapiens glucoside xylosyltransferase 1 (GXYLT1), transcript variant 1, mRNA [NM_173601]	NM_173601
HDHD2	Homo sapiens haloacid dehalogenase-like hydrolase domain containing 2 (HDHD2), mRNA [NM_032124]	NM_032124
HNF4G	Homo sapiens hepatocyte nuclear factor 4, gamma (HNF4G), mRNA [NM_004133]	NM_004133
HRH3	Homo sapiens histamine receptor H3 (HRH3), mRNA [NM_007232]	NM_007232
KIAA0319L (AU040320)	Homo sapiens KIAA0319-like (KIAA0319L), mRNA [NM_024874]	NM_024874
LAIR1	Homo sapiens leukocyte-associated immunoglobulin-like receptor 1 (LAIR1), transcript variant a, mRNA [NM_002287]	NM_002287
LIFR	Homo sapiens leukemia inhibitory factor receptor alpha (LIFR), transcript variant 2, mRNA [NM_002310]	NM_002310
LIN28B	Homo sapiens lin-28 homolog B (C. elegans) (LIN28B), mRNA [NM_001004317]	
MARK1	Homo sapiens MAP/microtubule affinity-regulating kinase 1 (MARK1), mRNA [NM_018650]	NM_018650
MATN3	Homo sapiens matrilin 3 (MATN3), mRNA [NM_002381]	NM_002381
MBD2	Homo sapiens methyl-CpG binding domain protein 2 (MBD2), transcript variant 2, mRNA [NM_015832]	NM_015832
MDH1B	Homo sapiens malate dehydrogenase 1B, NAD (soluble) (MDH1B), mRNA [NM_001039845]	NM_001039845
MFAP3L	Homo sapiens microfibrillar-associated protein 3-like (MFAP3L), transcript variant 1, mRNA [NM_021647]	NM_021647
MREG	Homo sapiens melanoregulin (MREG), mRNA [NM_018000]	NM_018000
MRO	Homo sapiens maestro (MRO), transcript variant 1, mRNA [NM_031939]	NM_031939
MSN	Homo sapiens moesin (MSN), mRNA [NM_002444]	NM_002444
NECAP1	Homo sapiens NECAP endocytosis associated 1 (NECAP1), transcript variant 1, mRNA [NM_015509]	NM_015509
NEK1	Homo sapiens NIMA (never in mitosis gene a)-related kinase 1 (NEK1), transcript variant 2, mRNA [NM_012224]	NM_012224
NEK6	Homo sapiens NIMA (never in mitosis gene a)-related kinase 6 (NEK6), transcript variant 2, mRNA [NM_014397]	NM_014397
NGFRAP1	Homo sapiens nerve growth factor receptor (TNFRSF16) associated protein 1 (NGFRAP1), transcript variant 3, mRNA [NM_014380]	NM_014380
NODAL	Homo sapiens nodal homolog (mouse) (NODAL), mRNA [NM_018055]	NM_018055
NPEPPS	Homo sapiens aminopeptidase puromycin sensitive (NPEPPS), mRNA [NM_006310]	NM_006310
OLFM1	Homo sapiens olfactomedin 1, mRNA (cDNA clone IMAGE:3351052), complete cds. [BC000189]	BC000189
OLFML2A	Homo sapiens olfactomedin-like 2A (OLFML2A), mRNA [NM_182487]	NM_182487
PAQR9	Homo sapiens progestin and adipoQ receptor family member IX (PAQR9), mRNA [NM_198504]	NM_198504
PEG10	Homo sapiens paternally expressed 10 (PEG10), transcript variant 1, mRNA [NM_001040152]	NM_001040152
PEX19	Homo sapiens peroxisomal biogenesis factor 19 (PEX19), transcript variant 1, mRNA [NM_002857]	NM_002857
PHLPP2	Homo sapiens PH domain and leucine rich repeat protein phosphatase 2 (PHLPP2), mRNA [NM_015020]	NM_015020
PPARG	Homo sapiens peroxisome proliferator-activated receptor gamma (PPARG), transcript variant 3, mRNA [NM_138711]	NM_138711
PPIF	Homo sapiens peptidylprolyl isomerase F (PPIF), nuclear gene encoding mitochondrial protein, mRNA [NM_005729]	NM_005729
PPIL6	Homo sapiens peptidylprolyl isomerase (cyclophilin)-like 6 (PPIL6), transcript variant 1, mRNA [NM_173672]	NM_173672
RCBTB1	Homo sapiens regulator of chromosome condensation (RCC1) and BTB (POZ) domain containing protein 1 (RCBTB1), mRNA [NM_018191]	NM_018191
RGPD5	Homo sapiens RANBP2-like and GRIP domain containing 5 (RGPD5), transcript variant 1, mRNA [NM_005054]	NM_005054
RPIA	Homo sapiens ribose 5-phosphate isomerase A (RPIA), mRNA [NM_144563]	NM_144563
SERPINA11	Homo sapiens serpin peptidase inhibitor, clade A (alpha-1 antiproteinase, antitrypsin), member 11 (SERPINA11), mRNA [NM_001080451]	NM_001080451
SFTPA1	Homo sapiens surfactant protein A1 (SFTPA1), transcript variant 1, mRNA [NM_005411]	NM_005411
SFXN4	Homo sapiens sideroflexin 4 (SFXN4), mRNA [NM_213649]	NM_213649
SLC30A10	Homo sapiens solute carrier family 30, member 10 (SLC30A10), mRNA [NM_018713]	NM_018713
SLC7A2	Homo sapiens solute carrier family 7 (cationic amino acid transporter, y+ system), member 2 (SLC7A2), transcript variant 2, mRNA [NM_001008539]	NM_001008539
TFPI	Homo sapiens tissue factor pathway inhibitor (lipoprotein-associated coagulation inhibitor) (TFPI), transcript variant 1, mRNA [NM_006287]	NM_006287
TMEM174	Homo sapiens transmembrane protein 174 (TMEM174), mRNA [NM_153217]	NM_153217
TMEM194B	Homo sapiens transmembrane protein 194B (TMEM194B), mRNA [NM_001142645]	NM_001142645
TMEM56	Homo sapiens transmembrane protein 56 (TMEM56), transcript variant 1, mRNA [NM_001199679]	NM_001199679
TMTC4	Homo sapiens transmembrane and tetratricopeptide repeat containing 4 (TMTC4), transcript variant 1, mRNA [NM_032813]	NM_032813
TSLP	Homo sapiens thymic stromal lymphopoietin (TSLP), transcript variant 1, mRNA [NM_033035]	NM_033035
TSPYL5	Homo sapiens TSPY-like 5 (TSPYL5), mRNA [NM_033512]	NM_033512
UAP1	Homo sapiens UDP-N-acteylglucosamine pyrophosphorylase 1 (UAP1), mRNA [NM_003115]	NM_003115
USP46	Homo sapiens ubiquitin specific peptidase 46 (USP46), transcript variant 1, mRNA [NM_022832]	NM_022832
VPS53	vacuolar protein sorting 53 homolog (S. cerevisiae) [Source:HGNC Symbol;Acc:25608] [ENST00000541903]	BC029560
XIRP1	Homo sapiens xin actin-binding repeat containing 1 (XIRP1), transcript variant 1, mRNA [NM_194293]	NM_194293
ZNF568	Homo sapiens zinc finger protein 568 (ZNF568), transcript variant 5, mRNA [NM_001204838]	NM_001204838

85 genes predicted as *miR-27b* targets by Trgetscan Human 7.0 were selected fromSupplementary Data set 2.

52 genes commonly predicted as *miR27b* target by Trgetscan Mouse 7.1 were highlighted in gray.

Supplementary Table 3 Validation of the target site conservation and gene expression for the 52 genes in Supplementary Table 2.

Gene	Suppressed below 0.6 in the three trials?	Target site-conservation?
AKIRIN	yes	yes
AQP11	no	yes
ARF3	nd	no
ASPH	nd	no
ATOH8	nd	no
B4GALT3	yes	yes
BRPF3	no	yes
C2CD2	nd	no
MAIP1	yes	yes
CAB39L	no	yes
CALM3	yes	yes
CCNG1	yes	yes
CHIC1	nd	no
CKAP4	yes	yes
DCP2	yes	yes
DDAH1	yes	yes
DPYD	no	yes
DOCK8	nd	no
EFNA5	not detected	yes
FAM69A	no	yes
GPR133	nd	no
GRM5	not detected	yes
GXYLT1	nd	no
HNF4G	nd	no
KIAA0319L	no	yes
LAIR1	nd	no
LIFR	no	yes
MARK1	no	yes
MATN3	no	yes
MREG	nd	no
MSN	not detected	yes
NECAP1	yes	yes
NEK1	no	yes
NEK6	yes	yes
NGFRAP1	yes	yes
NPEPPS	no	yes
OLFML2A	nd	no
PAQR9	no	yes
PHLPP2	yes	yes
PPARG	nd	yes
PPIF	no	yes
SERPINA11	nd	no
SFXN4	nd	no
SLC30A10	nd	no
SLC7A2	no	yes
TFPI	nd	no
TMEM174	nd	no
TMEM194B	no	yes
TMEM56	nd	no
TSPYL5	not detected	yes
USP46	no	yes
VPS53	nd	no

The 52 genes commonly predicted in Human and Mouse TargetScan were selected from the genes listed in Supplementary Table 2. Presence and absence of *miR-27b*-target sites conserved between *human* and *mouse* genomes are indicated as "yes" and "no" in the most right column, respectively. Genes with conserved sites were further analysed for expression in Figure 2b and those with downregulated expression below 0.6 compared to the control are indicated as "yes" in the middle column and ahownd in gray. The 4 genes were not detected and *PPARG* was not examined. nd, not done.

Supplementary Table 4 Oligonucleotides

qPCR primers	Forward primer	Reverse primer
GAPDH	GGTGGTCTCCTCTGACTTCAAC	GTGGTCGTTGAGGGCAATG
MARCH1	TGACAGGATTTCCCTTGCTC	CACCGTTAATCTAGCTCACTGC
AKIRIN	AGCTCCAACTTTGCATCCTG	AGGGCACAGCTGTTTATTGG
AQP11	TGACCCAGTATCACGTCAGC	TGACCGCTTTGAGCAAGTCG
ARF3	TCTGCCTAATGCTATGAACGC	ATGTACCAGTTACGGTGACGA
ASPH	GGGAGATTTTATTTCCACCTGGG	CCTTTGGCTTTATCCATCACTGC
B4GALT3	CGAGATCAGGGACCGACATTT	GATCGTTCTGGACAGTAGGGC
BRPF3	AGCTTCCGTATGGTGGACTCA	AGGTGGCTTCTCAATGTAGCG
C2CD2	GGTTCTGATTACCAAGCCCAC	GGATTCCTGAGCAGTAGATGC
MAIP1	CTACAGCACGGAGGAGAAGC	CGGAGAACTCTGTGATGCTG
CAB39L	GGCCTGCTAGTGACACTGATA	TACTCCACAGTAGGACTCCGA
CALM3	CAACACTCCCCTGCGTACC	GGGCATGGAGAGAGACTCAG
CCNG	CCTTCTGTGTTGGCATTGTC	AAGCTCTTGCCAGAAGGTCA
CKAP4	TGGACAGTTTGGTTGCATACTC	CCTCAGGTCATCTAGTAAACCCT
CORIN	AGGCCACTTTACGCAACTC	GCTGGTGGAGGTTAGTAGGG
DCP2	TAAAGCACAGGCAACCACTG	TCTGCCATTTCCCCTCATAC
DDAH	ACGAGGTGCTGAAATCTTGG	CCAATTGCGATCAGGTTAGG
DPYD	GGCGGACATCGAGAGTATCCT	TTCTTGGCCGAAGTGGAACAC
FAM69A	ATGGGCCTGCATGTAACAG	CTGATTGTTGGGCTTGGTG
GPR133	AAAGTCCCGGAGTGATACTGA	TTGGTGAGATTCAAGGCTGTC
HNF4G	GAGGTTCCAAGTGCAGATCG	GCAGGAGCAGAAGCAACTC
KIAA0319L	GGAGATCCTGTAGCCACCTG	GACTCGGGAGGTAGGAGGAC
LIFR	CACCTGGTCTTGCGAGCCTA	AGCCACTGCCACTGGGATGA
MARK1	GATGTGGCTCGAAAACTTGG	TTTCCTCTCTGGCCCTACAA
MATN3	TCTCCCGGATAATCGACACTC	CAAGGGTGTGATTCGACCCA
MBD2	CGAAAATCTGGGCTAAGTGC	GAGGATCGTTTCGCAGTCTC
MREG	GGACCGTCTCATTGCACTTGA	GGGACTTGGAAACGGAAGGTA
NECAP1	GGATGCTCGTCCTAAGTTGG	GAGGATGGTGGGGGAATAGT
NEK1	CAGTGATGTGGAGCCAACTG	AGTGCCATTCTTCGTTCAGG
NEK6	CAGGACTGTGTCAAGGAGATCG	ATGTTCAGCTCGTTGTCTTCG
NGFRAP1	GATGGGTGGAGATGGAGATG	GCAGGAGTCAAGGCATAAGG
NPEPPS	GTGAGGCAGGCGACTAATCAG	GTTCCCGTACCTGTTTGCAG
PAQR9	CAAGAGCCGTACCGACTGG	CCGATAATGTCGAAAAGACCCG
PHLPP2	TGGAACCTACTGAACGACCTC	ATCCAAACGATCCATGTGGCA
PLEKHJ1	CAGCTTCATTGAGGACCCTGA	CCTCGGATATGCCGAACTGTT
PPIF	TGAACCTGGGAACAAACCTC	GGAGCACAGGAGCTTACAGG
SFXN4	ACGCCACTGAAAGGGATCAAG	ACGGCTCCCGCCATTAGTA
SLC7A2	CCTTATGGCTTTACGGGAACG	CGAGGAGGTAGTACGGCATCA
TFPI	GATGGTCCGAATGGTTTCCAG	GATTGCGGAGTCAGGGAGTTA
TMEM194B	TCCCTGGTTCTGGTCTATGC	GCTCTCAGTGGGTAGTGCAG
TMEM56	TCCCTATCTGCTTTCCTTGC	TACTGATGGTGCTGGGTTCA
USP46	CACTATTGCGGACATCCTTCAG	CAGGTTCGTTCATGTTGCCA
VPS53	TCCCCTCAGTCCATTTTCTG	GCCAGCTTTCAGACAAGTCC
mouse B4galt3	ATGATGTATCTGTCACTAGGGGG	GGACCCACTAAGAAGGGTGAT
mouse Maip1	ATTCTGGTTCTCTGGCGTTG	CAGCCCTCGCTTCTTTACC
mouse Phlpp2	GGGGCAATGGTGGTTTATTTG	CAGTTGAAGCAAATCCCACAGG
mouse Gapdh	AGGTCGGTGTGAACGGATTTG	TGTAGACCATGTAGTTGAGGTCA
**3'UTR cloning primers**		
B4GALT3	CGACCTCGAGCACCTGGTTCCTCCCAAGCCTTCCG	GGTGGCGGCCGCAGAAAATGCCCAGGGAGGGGCTTC
MAIP1	CGACCTCGAGTCAGCTTATGGACTTTAGCAGTTG	GGTGGCGGCCGCGGTGATAACAATCTGTGGAAAATGC
PHLPP2	CGACCTCGAGCAGTCCTACCACCTGCCATT	GGTGGCGGCCGCCATCCCTCTCCTCCAGCATA
**Mutation primers**		
B4GALT3 seed-1 mutation	CTCCTGTAGGGCTTATGATTATAAATCCTTGATGTCATG	CATGACATCAAGGATTTATAATCATAAGCCCTACAGGAG
B4GALT3 seed-2 mutation	GAGTGATCTGGCTTCTCCTCGGACCTTTATAAATATTTATTC	GAATAAATATTTATAAAGGTCCGAGGAGAAGCCAGATCACTC
MAIP1 seed-1 mutation	GGACTTTAGCAGTTGTTATAAAAAACTAAGGAAGAAAAATTTTGGG	CCCAAAATTTTTCTTCCTTAGTTTTTTATAACAACTGCTAAAGTCC
MAIP1 seed-2 mutation	CACTTAATCTAAGTTTATAAATTACTTTTATATTATTTTGAAATACTCC	GGAGTATTTCAAAATAATATAAAAGTAATTTATAAACTTAGATTAAGTG
PHLPP2 seed-1 mutation	GAGCACTTTTCATTGATTATAAACTTTTTATTTTTGAATCTGCACTCG	CGAGTGCAGATTCAAAAATAAAAAGTTTATAATCAATGAAAAGTGCTC
PHLPP2 seed-2 mutation	CGAGGGTATAGGAAGGGCCTTATAAAGTTGATTTAACTTTTGG	CCAAAAGTTAAATCAACTTTATAAGGCCCTTCCTATACCCTCG
PHLPP2 seed-3 mutation	GTTGATTTAACTTTTGGATGTCAGATTATAAAAGCTCCTGAGAAACTTGG	CCAAGTTTCTCAGGAGCTTTTATAATCTGACATCCAAAAGTTAAATCAAC
**ORF cloning primers**		
B4GALT3	GGTGGCGGCCGCCGGTTTCATGATTAAGGTAGAC	CGATCTAGAGGAGTGCTGGATGGAGCCTTTCTCTGCC
MAIP1	ATTTGCGGCCGCGGGTCATAAAAATGGCGCTGG	CGCGTCTAGAAGTCCATAAGCTGATTTTTCC
**TK promoter cloning primers**	TCGAACCGGTCCGTGGCCCGTTGCTCGCGTTTG	TCGAGCGGCCGCTAAGCGGGTCGCTGCAGGGTCGC
