# Supplementary material for: miR-27b targets MAIP1 to mediate lipid accumulation in cultured human and mouse hepatic cells
Source: Commun Biol. 2023 Jun 24;6:669. doi: 10.1038/s42003-023-05049-w (PMC10290684; doi:10.1038/s42003-023-05049-w)
Supplement: Supplementary file 3 — Description of Additional Supplementary Files [file 42003_2023_5049_MOESM3_ESM.pdf]

Description of Additional Supplementary Files

File name: Supplementary Data 1

Description: Genes with fold change cut-offs of 2< (miR-27b vs contol) in microarray analysis.

File name: Supplementary Data 2

Description: Genes with fold change cut-offs of < -2 (miR-27b vs contol) in microarray analysis.

File name: Supplementary Data 3

Description: The source data behind the graphs in the paper.
