# Supplementary material for: miR-27b targets MAIP1 to mediate lipid accumulation in cultured human and mouse hepatic cells
Source: Commun Biol. 2023 Jun 24;6:669. doi: 10.1038/s42003-023-05049-w (PMC10290684; doi:10.1038/s42003-023-05049-w)
Supplement: Supplementary file 7 — Reporting Summary [file 42003_2023_5049_MOESM7_ESM.pdf]

nature portfolio

Corresponding author(s): Hiroyuki Mizuguchi
Last updated by author(s): Jun 8, 2023

Reporting Summary

Nature Portfolio wishes to improve the reproducibility of the work that we publish. This form provides structure for consistency and transparency in reporting. For further information on Nature Portfolio policies, see our Editorial Policies and the Editorial Policy Checklist.

Statistics

For all statistical analyses, confirm that the following items are present in the figure legend, table legend, main text, or Methods section.

n/a	Confirmed	
☐	☒	The exact sample size (*n*) for each experimental group/condition, given as a discrete number and unit of measurement
☐	☒	A statement on whether measurements were taken from distinct samples or whether the same sample was measured repeatedly
☐	☒	The statistical test(s) used AND whether they are one- or two-sided<br>*Only common tests should be described solely by name; describe more complex techniques in the Methods section.*
☒	☐	A description of all covariates tested
☒	☐	A description of any assumptions or corrections, such as tests of normality and adjustment for multiple comparisons
☐	☒	A full description of the statistical parameters including central tendency (e.g. means) or other basic estimates (e.g. regression coefficient) AND variation (e.g. standard deviation) or associated estimates of uncertainty (e.g. confidence intervals)
☐	☒	For null hypothesis testing, the test statistic (e.g. *F*, *t*, *r*) with confidence intervals, effect sizes, degrees of freedom and *P* value noted<br>*Give P values as exact values whenever suitable.*
☒	☐	For Bayesian analysis, information on the choice of priors and Markov chain Monte Carlo settings
☒	☐	For hierarchical and complex designs, identification of the appropriate level for tests and full reporting of outcomes
☐	☒	Estimates of effect sizes (e.g. Cohen's *d*, Pearson's *r*), indicating how they were calculated

Our web collection on statistics for biologists contains articles on many of the points above.

Software and code

Policy information about availability of computer code

Data collection	Not applicable
Data analysis	Not applicable

For manuscripts utilizing custom algorithms or software that are central to the research but not yet described in published literature, software must be made available to editors and reviewers. We strongly encourage code deposition in a community repository (e.g. GitHub). See the Nature Portfolio guidelines for submitting code & software for further information.

Data

Policy information about availability of data

All manuscripts must include a data availability statement. This statement should provide the following information, where applicable:

- Accession codes, unique identifiers, or web links for publicly available datasets
- A description of any restrictions on data availability
- For clinical datasets or third party data, please ensure that the statement adheres to our policy

Data from microarray analysis have been deposited in GEO under accession number GSE174075. Accesion code for GSE174075 is obkxakwovbcxvcv.

Human research participants

Policy information about studies involving human research participants and Sex and Gender in Research.

Reporting on sex and gender	not applicable
Population characteristics	not applicable
Recruitment	not applicable
Ethics oversight	not applicable

Note that full information on the approval of the study protocol must also be provided in the manuscript.

Field-specific reporting

Please select the one below that is the best fit for your research. If you are not sure, read the appropriate sections before making your selection.

☒ Life sciences ☐ Behavioural & social sciences ☐ Ecological, evolutionary & environmental sciences

For a reference copy of the document with all sections, see nature.com/documents/nr-reporting-summary-flat.pdf

Life sciences study design

All studies must disclose on these points even when the disclosure is negative.

Sample size	No sample-size calculations were performed.
Data exclusions	No data were excluded.
Replication	All experimental results were confirmed reproducible.
Randomization	All samples were allocated randomly.
Blinding	Blinding was not performed.

Reporting for specific materials, systems and methods

We require information from authors about some types of materials, experimental systems and methods used in many studies. Here, indicate whether each material, system or method listed is relevant to your study. If you are not sure if a list item applies to your research, read the appropriate section before selecting a response.

Materials & experimental systems

n/a	Involved in the study	
☐	☒	Antibodies
☐	☒	Eukaryotic cell lines
☒	☐	Palaeontology and archaeology
☐	☒	Animals and other organisms
☒	☐	Clinical data
☒	☐	Dual use research of concern

Methods

n/a	Involved in the study	
☒	☐	ChIP-seq
☒	☐	Flow cytometry
☒	☐	MRI-based neuroimaging

Antibodies

Antibodies used	Anti-G3PDH poiyclonal antibody (2275-PC-100, Trevigen); anti-MAIP1 polyclonal antibody (HPA064013, Atlas Antibodies); anti-PHLPP2 polyclonal antIBODY (ab71973, Abcam); anti-B4GALT3 polyclonal antibody (HPA010793, Atlas Antibodies); anti-AMPK polyclonal antibody (23H3, Cell Signaling); anti-phosphorylated AMPK polyclonal antibody (40H9, Cell Signaling Technology)
Validation	All antibodies were validated for western blotting in the manufacturer's web sites. Also, anti-MAIP1. anti-PHLPP2 and anti-B4GALT3 antibodies were validated by the knockdown experiments for each gene.

Eukaryotic cell lines

Policy information about cell lines and Sex and Gender in Research

Cell line source(s)	Huh-7 and HepG2 cell lines were obtained from JCRB Cell Bank, Tokyo, Japan. 293T cell line was from an unknown origin but has been used for virus preparation in our lab.
Authentication	Huh-7 and HepG2 cell lines have been authenticated by JCRB Cell Bank.
Mycoplasma contamination	All cell lines were tested negative for mycoplasma contaminations.
Commonly misidentified lines (See ICLAC register)	none

Animals and other research organisms

Policy information about studies involving animals; ARRIVE guidelines recommended for reporting animal research, and Sex and Gender in Research

Laboratory animals	4-9weeks old C57B/6N and 6 weeks old C57B/6J
Wild animals	none
Reporting on sex	none
Field-collected samples	none
Ethics oversight	All procedures were approved by the Osaka University Institutional Animal Care and Use Committee.

Note that full information on the approval of the study protocol must also be provided in the manuscript.
